# Exploring Perspectives on Kidney Donation: Medical and Non-Medical Students in Croatia

**DOI:** 10.3390/jcm15020681

**Published:** 2026-01-14

**Authors:** Ariana Tea Šamija, Lara Lubina, Victoria Frances McGale, Nikolina Bašić-Jukić

**Affiliations:** 1 School of Medicine, University of Zagreb, 10000 Zagreb, Croatia; lara.lubina@gmail.com (L.L.); victoriamcgale1@gmail.com (V.F.M.); nikolina.basic.jukic@kbc-zagreb.hr (N.B.-J.); 2 Department of Internal Medicine, University Hospital Center Zagreb, 10000 Zagreb, Croatia

**Keywords:** public health, kidney donation, transplantation in Croatia, university students

## Abstract

**Background/Objectives:** Kidney donation remains a critical component of addressing end-stage renal disease. This study examines differences in awareness, willingness to donate, and concerns related to kidney donation among medical and non-medical university students. By comparing these groups within the context of Croatia’s presumed-consent system for organ donation, the study provides insights into how educational backgrounds shape attitudes in a setting with high transplantation rates but limited data on young adults. **Methods:** A cross-sectional observational study targeted at medical and non-medical university students in Croatia. Data were collected from 640 participants via a self-administered, close-ended, structured questionnaire with 33 items divided across three sections. Responses were analyzed using IBM SPSS Statistics program (v. 30.0), to identify significant differences. Due to the cross-sectional design, causal relationships could not be inferred. **Results:** Overall, 190 students (28.7%) reported willingness to donate a kidney during their lifetime, which was more common among medical students (*N* = 59; 39.0%) than non-medical students (*N* = 131; 26.8%). Collectively, willingness to donate postmortem was high in both groups (*N* = 527; 82.3%), as was willingness in a brain-dead state (*N* = 448; 70.0%). Medical and non-medical students mostly cited perceived health risks as a concern and concerns related to surgical complications. Regarding information sources, 33.2% of students reported inadequate knowledge of kidney donation, with social media and internet searches cited more frequently than healthcare professionals. **Conclusions:** Our findings indicate that medical and non-medical students exhibit distinct gaps in knowledge, risk perception and willingness toward kidney donation. Within Croatia’s presumed-consent framework, these findings highlight the importance of targeted educational strategies to support informed decision-making among future generations.

## 1. Introduction

Chronic kidney disease (CKD) affects more than 800 million people globally and as of 2024, is the 9th leading cause of death worldwide [1,2]. In Croatia, it is estimated that 12.7% of the population has newly discovered or previously diagnosed CKD [3]. By the end of 2020, the incidence of kidney replacement therapy (KRT) was 109.3 per million population [4] (Table C.2.7, p. 102). The high prevalence of kidney disease treatment emphasizes a significant burden on healthcare institutions. The need for renal replacement therapy is expected to persist or grow as Croatia’s population ages and as chronic illnesses remain prevalent [5]. This highlights end-stage renal disease (ESRD) as a critical health issue that requires urgent attention.

Patients with ESRD have various treatment options available to them based on the stage of their illness. While there are different dialysis types offered, the preferred treatment method is kidney transplantation, as it improves both the lifespan and quality of life [6]. Nevertheless, kidney transplantation is not always feasible due to a significant shortage of organs. Based on Eurotransplant data, as of 2024, there were approximately 270 patients actively waiting for a kidney transplant in Croatia [7]. This figure reflects the number of people striving for better health outcomes, while enduring frequent and exhausting treatments.

Since performing its first kidney transplant in Rijeka in 1971, Croatia has made significant progress in the field of kidney transplantation. Many factors contributed to this successful journey, notably the fact that for the past 37 years, Croatia has been utilizing the so-called “presumed consent” system of organ donation. This method presumes automatic consent for organ donation unless an individual has officially registered their refusal. This allowed Croatia to position itself as one of the leading European nations in the field of transplantation [8].

According to Eurotransplant data, in 2024 Croatia performed 36.3 per million population (pmp) kidney transplants from deceased donors and 0.8 pmp kidney transplants from living donors [7]. Regarding deceased donor transplants, Croatia is above the other Eurotransplant program member countries, which average 23.1 transplants pmp. Conversely, Croatia demonstrates a lower rate of living kidney donor transplants than the other Eurotransplant member countries, with an overall average rate of 9.5 pmp.

Croatia’s success in organ transplantation can be attributed to the legislation policies implemented in April of 1988 [8]. A “presumed consent” organ donation system was implemented, in which all deceased individuals are eligible to become organ donors unless they had formally registered an objection during their lifetime. The integration of national policy with efficient coordination among transplant centers has established Croatia as a leading European country in kidney transplantation, reflected in its high deceased donor transplant rates and strong Eurotransplant foundation collaboration [9]. Nonetheless, it is vital to maintain this success and continue to expand it in the future years.

Medical and non-medical students’ willingness to donate a kidney is significantly influenced by background knowledge, beliefs and attitudes [10]. In Croatia there is a lack of research on university students’ attitudes toward organ donation. Medical students are an important part of the community, as future healthcare providers they will be responsible for guiding, influencing and educating public attitudes toward kidney donation and transplantation. Medical students represent future healthcare providers which will shape public knowledge and attitudes, whereas non-medical students provide insight into how kidney donation will be perceived, understood and supported in the future broader population, outside of a medical setting.

The effectiveness of this organ donation system relies heavily on public awareness and acceptance. Given the fundamental roles of healthcare professionals and the general public, medical and non-medical university students’ perception is crucial for evaluating and predicting future donation trends. Understanding the outlooks of future healthcare providers is vital, as they will play a critical role in educating and influencing public opinion on kidney donation. Additionally, examining non-medical students’ views offers insight into the knowledge and acceptance of kidney donation among a younger demographic, who will inherit the burden of organ donation within Croatia’s unique legislative framework. This comparative approach allows us to identify potential gaps in awareness and misconceptions that may exist in both groups. By highlighting the differences and similarities in attitudes, we can better understand the impact of medical education on kidney donation awareness and explore opportunities to strengthen public education initiatives.

From a theoretical perspective, educational background may influence kidney donation behaviors through its effects on knowledge acquisition, risk perception and attitudes towards transplantation. Greater exposure to medical education is expected to be associated with higher procedural knowledge and more specific risk assessment. Limited formal education or healthcare exposure may contribute to lack of knowledge on the reality of kidney transplantation and donation procedures. This leads to generalized health related fears and misconceptions with consequently lower willingness to donate.

Our aim was to assess medical and non-medical students in terms of the gaps and differences in their knowledge, attitudes, and willingness to donate their kidneys and to investigate how different educations affect these views. This is especially important in countries such as Croatia, where high transplantation rates coexist with limited data on young adults’ perspectives, who will constitute future societal attitudes towards organ donation.

## 2. Materials and Methods

This cross-sectional observational study aimed to analyze the willingness toward kidney donation comparing Croatian medical and non-medical students. Participants were required to be at least 17 years of age, currently enrolled in a Croatian university, and of either gender. The sample was divided into two main groups:

- Medical students (*N* = 151): Students enrolled in a Croatian university medical degree program (Doctor of Medicine).
- Non-medical students (*N* = 489): Students enrolled in non-health-related disciplines, including engineering, social sciences, humanities, and other fields.

All participants completed a self-administered, structured questionnaire designed to assess their willingness toward kidney donation. The questionnaire consisted of close-ended items to ensure reliability and consistency. The unequal sample size reflects the voluntary nature of the survey participation and the broader enrollment of non-medical disciplines at Croatian universities. The questionnaire was distributed online via university platforms and social media channels. Initially, the survey link was shared through university group chats and social media platforms. Various Croatian university professors were also contacted to promote the survey within their respective student groups. Although recruitment efforts were concentrated in Zagreb, participation was open to students nationwide. The study protocol was approved by the University of Zagreb Ethics Committee (Approval No. 251-59-10106-25-111/286). Informed consent was obtained electronically from all participants before completing the questionnaire. Participation was voluntary and anonymous.

Data collection was conducted in 2024, from the end of January to mid-February. The questionnaire used in this study was structured into three main sections to comprehensively assess participants’ demographics, knowledge, and attitudes toward kidney donation. The observed internal consistency of the attitude and belief scale (Cronbach’s α = 0.69) reflects the inclusion of multiple interrelated yet distinct dimensions of kidney donation, including willingness, perceived risk, ethical considerations, familial influences, physician endorsement and attitudes towards financial incentives, rather than a single homogeneous construct within this exploratory study. Minor cultural adaptations were made to ensure its relevance to the Croatian student population. Given the use of heterogeneous response formats across the items, the questionnaire is described narratively to accurately reflect its structure. In total, the questionnaire comprised 33 items:

- Eight items assessing demographics;
- Seven items measuring awareness and knowledge;
- Eighteen items exploring attitudes and beliefs.

The first section collected demographic data about the participants. It included eight items capturing essential background information such as age, gender, academic discipline, year of study, and prior exposure to organ donation education. These items include:

- Gender.
- Age.
- Education level (Multiple choice).
- Are you a medical or non-medical student? (Multiple choice)
- Religious affiliation (Multiple choice).
- Do your religious or cultural beliefs influence your views on kidney donation? (Yes or No question)
- Do you have any known chronic health conditions? (Yes or No question)
- Do you have a family history of kidney disease? (Yes or No question)

The second section assessed participants’ awareness and knowledge of kidney donation. This section consisted of seven items, including six multiple-choice questions and one item using a five-point Likert scale. The Likert scale ranged from “strongly agree” to “strongly disagree,” allowing participants to express their level of agreement with statements related to kidney donation knowledge. The questions from this section include:

- How did you learn about organ donation in Croatia? (Multiple choice)
- Did you know that in Croatia every citizen is by default an organ donor, unless they opt out? (Multiple choice)
- What is your view on the fact that Croatia has a default organ-donor system, where all citizens are considered organ donors unless they opt out? (Likert scale)
- Have you or someone you know donated or received a kidney organ? (Multiple choice)
- Who do you ethically consider a suitable kidney organ donor? (Multiple choice)
- Success rate of kidney transplant in patient after 1 year? (Percentages)
- A healthy person can survive with only one functioning kidney. (True or false)

The third section explored participants’ attitudes and beliefs regarding kidney donation. It included one multiple-choice question, three yes or no question and fourteen items using the same five-point Likert scale as in the previous section. These items aimed to capture participants’ opinions, perceptions, and willingness toward kidney donation. The statements include:

- I would be more willing to donate my kidney if I had more information about the kidney transplantation process and consequences. (Likert scale)
- I would be more willing to donate my kidney if a person I knew required a kidney transplant. (Likert scale)
- I would only potentially donate my kidney to a loved one or family member. (Likert scale)
- I would potentially donate my kidney to an unrelated person in need. (Likert scale)
- Would you consider donating a kidney while alive? (Yes or No question)
- I would consider donating my kidney if I was in a coma/brain-dead. (Likert scale)
- Are you willing to donate your kidneys after your death? (Yes or No question)
- My concerns (if any) around kidney donation. (Multiple choice)
- I am worried about my family’s approval and objection regarding donating my kidney. (Likert scale)
- Would you ever encourage your family, friends, or peers to consider kidney donation? (Yes or No question)
- My attitude towards living volunteer living kidney donation. (Likert scale)
- My attitude towards ‘brain-dead’ person kidney organ harvesting. (Likert scale)
- My attitude towards deceased volunteer kidney organ donation. (Likert scale)
- I would consider donating my kidney for a sum of money/reward. (Likert scale)
- I am worried that kidney transplantation will leave me weak or disabled. (Likert scale)
- Donating your kidney impacts your life in a negative way. (Likert scale)
- Donating my kidney impacts my life in a positive way. (Likert scale)
- Kidney donations should be promoted by your physician. (Likert scale)

To ensure consistency and clarity, the term brain death is used to denote the irreversible cessation of all brain function; where the term coma appeared in the questionnaire, it was interpreted within this specific clinically defined context, given its relevance to organ donation practices. The survey was administered via Google Forms, with settings which enabled only one response per participant. All data points were stored securely in encrypted storage, accessible only to the principal authors. Prior to analysis, data were reviewed for completeness and screened for duplicate or invalid responses to ensure data quality.

Data were analyzed using SPSS Statistics version 30.0 (IBM Corp., Armonk, NY, USA). Descriptive statistics were expressed as frequencies and percentages for categorical variables, while quantitative data were summarized as mean ± standard deviation for normally distributed variables and median (interquartile range) for non-normally distributed data. Inferential analyses included chi-square tests for categorical variables and Mann–Whitney U tests for ordinal (Likert-scale) responses. Independent samples *t*-tests were used for normally distributed quantitative variables. Statistical significance was set at *p* < 0.05. A formal sample size calculation was not performed; however, the total sample size of 640 participants was considered adequate based on the exploratory nature of the study and the questionnaire-based design, allowing meaningful descriptive and comparative analyses between medical and non-medical students.

## 3. Results

### 3.1. Characteristics of Respondents

The study population consisted of 640 Croatian university students. The participants were divided into two groups: the first group consisted of medical students (*N* = 151; 23.6%) and the second group consisted of non-medical students (*N* = 489; 76.4%). Studying the baseline characteristics of the entire sample, most participants (66.5%) were aged between 18–21 years, the dominant gender was female (*N* = 457; 71.1%) and all participants have completed secondary education prior to enrollment into their respective universities.

Pertaining to medical students specifically, most participants (*N* = 120; 79.5%) were in their preclinical years (1st–3rd year), followed by participants (*N* = 31; 20.5%) in their clinical years (4th–6th year). Among non-medical students, the most enrolled university program was an undergraduate degree (bachelor’s degree level), with 430 students (87.9%), followed by postgraduate programs (master’s and doctorate level), with 59 students (12.1%). Summarized demographic data of the two groups are shown in Table 1.

### 3.2. Contextual Factors Influencing Kidney Donation Beliefs

To better understand potential influences on participants’ views toward kidney donation, there was a preemptive assessment of factors such as religious or cultural beliefs, chronic health conditions, family history of kidney disease and personal experience with kidney donation, with detailed results displayed in Table 2.

Majority of medical and non-medical students reported that their religious and cultural beliefs did not influence their views on kidney donation (*N* = 506; 79.0%).

Regarding individual and familial health conditions, the majority (*N* = 564; 88.1%) reported not having a chronic health condition and 70.3% (*N* = 450) of students denied family history of kidney disease. Most students (83.1%) did not know someone who had donated or received a kidney. Of the 80 participants which confirmed family history of kidney disease, 10.4% were non-medical students (*N* = 67) and 8.6% were medical students (*N* = 13). A total of 110 participants (17.2%) indicated that they were unsure whether they had a family history of kidney disease and 11.0% of participants (*N* = 71) confirmed that they had a chronic health condition.

### 3.3. Awareness and Knowledge Regarding Kidney Donation

**Figure 1:**
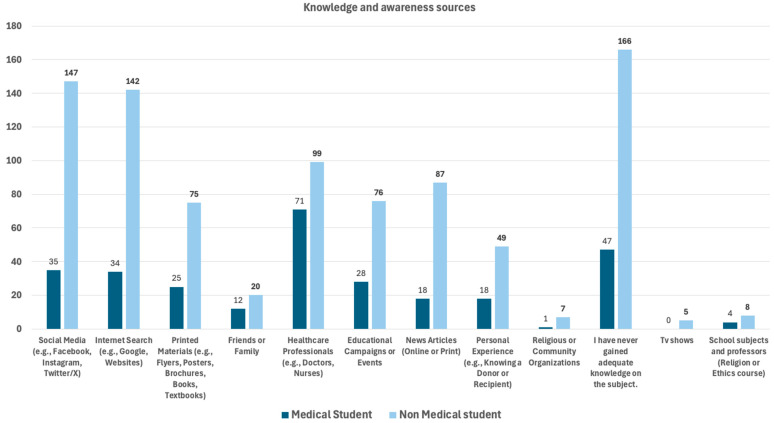
Sources of kidney donation knowledge and awareness as reported by medical and non-medical students.

Knowledge sources and awareness of kidney transplantation and donation across both student groups are shown in Table 3. To assess where participants acquired their knowledge about the topic, they were presented with a multiple-choice question allowing multiple selections. The distribution of responses is illustrated in Figure 1. Non-medical students most frequently reported lack of adequate knowledge on the subject, with 166 (33.9%) students choosing this option. This was followed by social media platforms (*N* = 147; 30.1%), internet searches (*N* = 142; 29.0%), news articles (*N* = 87; 17.8%), and healthcare professionals (*N* = 99; 20.2%) as common sources of awareness about kidney donation.

In contrast, medical students most cited healthcare professionals (*N* = 71; 47.0%) as their primary source of information. This was followed by those who reported having never gained adequate knowledge on the topic (*N* = 47; 31.1%), social media (*N* = 35; 23.2%), internet searches (*N* = 34; 22.5%) and educational campaigns or events (*N* = 28; 18.5%).

These findings suggest that while non-medical students are more reliant on informal and online sources such as social media and news outlets, medical students primarily gain knowledge through formal or professional channels, particularly from healthcare professionals and educational initiatives. Notably, a substantial proportion of both groups indicated a lack of adequate knowledge on the subject, highlighting a gap in understanding that may warrant targeted educational efforts for this specific population.

A significant knowledge gap and difference in attitudes were observed regarding Croatia’s organ donation legislation, as depicted in Table 4 and Table 5. Majority of medical students (*N* = 83; 55.0%) and non-medical students (*N* = 389; 79.6%) were not aware of the current legislation concerning transplantation and organ donation in Croatia, where every Croatian citizen is by default an organ donor unless they opt out [8]. A significantly higher proportion (*p* < 0.001) of medical students (45.0%) were familiar with Croatia’s organ donation system compared to non-medical students (20.4%).

Medical students had significantly more favorable attitudes (80.1% agreeing or strongly agreeing) toward Croatia’s default organ-donor system than non-medical students (*p* < 0.001). This difference is likely due to medical students’ exposure to education on organ donation and its clinical relevance, throughout their studies.

While non-medical students tended to express moderate agreement (61.5% in total) or neutrality (22.3%) toward the default organ-donor system, medical students were more polarized in their responses. As seen in Table 5, a higher proportion of medical students agreed (19.9%) or strongly agreed (60.2%) with the system, suggesting a more divided stance amid the student groups (*p* = 0.001).

**Figure 2:**
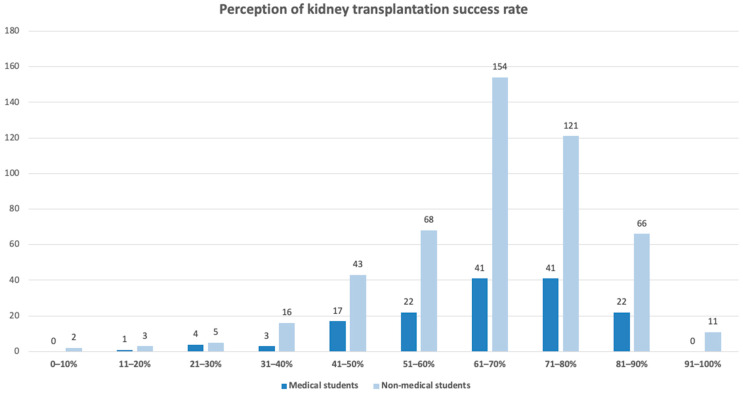
Perception among the two groups on kidney transplantation success rate (within 1 year of receiving organ).

According to most sources, the approximate global one-year graft survival rate after kidney transplantation is around 95.0% for recipients of deceased donor kidneys and approximately 97.8% for recipients of living donor kidneys [11,12]. Hence, the correct response category for this item was set to 91.0–100.0%. Figure 2 shows both groups’ perception of the success rate for kidney transplantation within the first year after receiving the organ. Notably, none of the medical students selected the correct response range, in comparison to only 11 non-medical students (2.3%) who selected the correct range.

Most participants underestimated the true success rate of transplantation, with responses mostly clustering between 61.0–80.0%. Although this finding may suggest limited awareness of current transplantation outcomes, it should be interpreted with caution. The absence of correct responses among medicals students and the minority of non-medical students selecting the correct response, may reflect ambiguity in question wording or interpretation. Additionally, students may have been uncertain whether the question referred to ‘success’ as graft versus patient survival or living versus deceased donor transplantation. The lack of statistically significant difference between groups (*p* = 0.466) further supports the need for cautious interpretation.

Medical students demonstrated higher awareness of basic organ function with 95.4% (*N* = 144) knowing a person could survive with a single functioning kidney [13], compared to 82.2% of non-medical students (*N* = 402, *p* < 0.001). As indicated in Table 6, a significantly higher proportion of non-medical students selected “Not sure” (15.3% vs. 2.6% of medical students), indicating a discrepancy in basic knowledge of kidney function.

### 3.4. Attitudes and Beliefs Towards Kidney Donation

Willingness to encourage others to donate has been tied to a medical educational background, since medical students showed more willingness to encourage their family, friends and peers to consider kidney donation, as seen in Table 7. Regarding physician-led promotion, both groups exhibited a high degree of neutrality, suggesting either limited awareness or a lack of strong opinion on this matter (Table 8). Physicians play a crucial role in informing patients about the possibility of organ donation and providing guidance on how to obtain a donor card or register their decision to opt out. The observed neutrality among student may be influenced by Croatia’s “presumed-consent” legislation, many of them may not perceive additional physician-led promotion as necessary.

**Figure 3:**
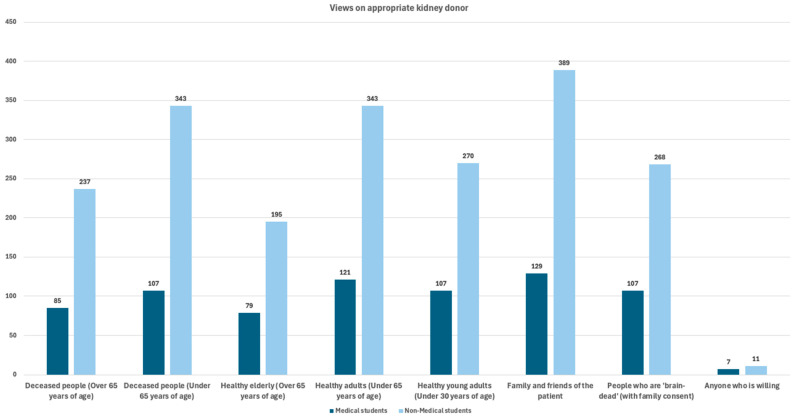
Views among medical and non-medical students on who is considered an ethically appropriate kidney donor.

Scientific evidence indicates living kidney donors under the age of 60 have the most favorable outcomes for both donor and recipient, and therefore ethically represent the optimal donor age group in clinical kidney transplantation studies [14]. Both groups, for the most part, primarily identified family and friends of the patient, healthy adults under 65 years of age, and deceased individuals under 65 years of age as the most ethically appropriate candidates for kidney donation. These results suggest a shared ethical preference for younger and healthier donors. The various replies are illustrated in Figure 3 and shown in Table 9.

In general, a positive perception on kidney donors was observed in both groups, showcasing a shared appreciation for the altruistic nature of organ donation. However, non-medical students appear to have more favorable views toward living volunteers than medical students (mean rank 333.2 and 279.2, respectively, *p* < 0.003), as shown in Table 10.

### 3.5. Willingness to Participate in Kidney Donation

Table 11 summarizes the willingness of medical and non-medical students to donate their kidneys in various scenarios, highlighting differences in agreement across a range of ethical and personal considerations. Both groups were in support that more information regarding kidney donation would increase their willingness to donate. Notably, this may reflect an area where targeted education could shift perceptions. The emotional factor appears to play a significant role in the high levels of willingness to donate a kidney to someone a student knows personally whether an acquaintance or a loved one.

In contrast, the significantly lower agreement observed in responses regarding the willingness to donate to an “unrelated person in need” highlights the crucial role of emotional and interpersonal connections. These findings suggest that such personal ties are a key driving factor influencing students’ willingness to consider kidney donation.

Over half of medical students (*N* = 86; 57.0%) were willing to donate in the event of brain death, highlighting the key understanding of posthumous organ donation within the medical community. This ethical responsibility of an individual’s end-of-life donation was also expressed within the non-medical group. In the instance of brain death, 235 non-medical students (48.1%) strongly agreed to donate their kidneys. Medical students are less supportive of monetary incentives for kidney donation than non-medical students (*p* = 0.011). Medical and non-medical students showed a generally altruistic attitude toward kidney donation, especially for known individuals or in case of brain death. These findings can serve as a basis for future public health initiatives.

Table 12 presents the willingness of students to donate a kidney either during their lifetime or after death. A significantly higher proportion of medical students (39.1%) expressed willingness to donate a kidney while alive compared to non-medical students (26.8%, *p* = 0.014). This finding suggests that medical students may possess a greater understanding of the safety and ethical acceptability of living kidney donation. Nevertheless, the majority of both groups remain undecided regarding living donation, with 59.1% of non-medical students and 50.3% of medical students reporting uncertainty. Targeted public health initiatives aimed at improving knowledge and addressing misconceptions could help increase willingness among students who have not yet formed a concrete decision on the matter.

In contrast, when asked about posthumous kidney donation, both groups showed similarly high levels of agreement, with 86.1% of medical students and 81.2% of non-medical students willing to be post-mortem donors. The shared willingness among both groups possibly reflects a broader generational norm or belief in contributing to others well-being, especially when the risks are negligible (posthumous donation).

### 3.6. Concerns and Barriers of Kidney Donation

Outlined in Table 13 are the various concerns students reported regarding kidney donation. The most cited concern in both groups was “health risks”, with 76.8% of medical students and 78.7% of non-medical students selecting this option. The shared perception of the potential “health risks” of transplantation can be a barrier for future kidney donation and should be addressed in future public health initiatives.

Concerns of potential “surgical complications” were also widely shared; 72.9% of medical students and 75.7% of non-medical students expressed this concern. A statistically significant difference was observed (*p* = 0.001) regarding the concern for “lack of compensation or financial support”, which was reported by 21.9% of non-medical students compared to only 9.9% of medical students. Mitigation of these concerns may be achieved through the introduction of ethically appropriate, non-monetary support mechanisms, such as reimbursement of documented expenses (such as travel costs, lost wages, accommodation, and post-donation medical care) and formal social acknowledgment of donors (certificates and entry into the national donor registry).

Another concern, such as the “effect kidney donation has on a person’s future”, ranked highly across both groups (54.3% of medical students and 47.6% of non-medical students) which shows that many students, regardless of academic background consider the long-term personal and professional implications of donation as a significant factor in their decision-making process. To mitigate students’ fears that kidney donation could negatively impact their health or quality of life, it is essential to strengthen educational and informational efforts through evidence-based communication of long-term donor outcome data.

As seen in Table 14, most students expressed uncertainty about whether kidney donation would impact their lives positively, with the majority selecting neutral responses (*N* = 347; 54.3%, in total).

Non-medical students, however, were slightly more inclined to believe in potential negative effects, though this was not statistically significant. Interestingly, the only significant difference was seen in the concern for “family approval”, where medical students were more concerned than non-medical students (*p* = 0.044). This implies that familial influence and expectations may impact groups differently and should be explored further.

Overall, while both groups show mixed feelings, no major fear-based barrier stands out apart from general uncertainty, highlighting the importance of education to clarify the real-life impact of donation.

## 4. Discussion

This study aimed to compare medical and non-medical university students in Croatia with respect to their gaps in knowledge, attitudes and willingness towards kidney donation. The findings demonstrate high overall willingness for posthumous donation in both groups, alongside lower willingness for living donation, particularly among non-medical students. These results highlight the need for targeted public health initiatives if Croatia is to improve its rates of living kidney donation. Non-medical students generally reported uncertainty and lack of knowledge regarding the consequences of kidney donation and were more concerned with financial support. Medical students identified their major concerns as being a perceived health risk and possible surgical complications.

Additionally, medical students exhibited higher awareness of Croatia’s organ donation legislation and were more accepting of the country’s “presumed consent” system compared to non-medical students. Taken together, the findings of this study suggest a conceptual pathway linking educational exposure, perceived risk, and willingness to donate a kidney. Greater exposure to medical education may enhance understanding of transplantation procedures and outcomes, which in turn may reduce uncertainty and perceived risk, thereby supporting willingness to donate. Conversely, limited educational exposure may contribute to knowledge gaps, heightened uncertainty, and lower willingness, particularly with respect to living donation. Importantly, these associations should be interpreted as exploratory and descriptive rather than causal.

### 4.1. Willingness to Donate Kidneys: Alive vs. Postmortem

Both medical and non-medical students in our study reported high willingness to donate kidneys after death, but medical students were significantly more willing to donate while alive. Formal medical education may have contributed to a better understanding of the safety of living kidney donation, fostering confidence that donors can continue to live normal, healthy lives.

Comparison with international literature is constrained by the limited number of questionnaire-based studies conducted among university-aged populations. Nevertheless, our results align with some international studies examining medical students’ willingness to become living kidney donors. A Spanish study by Ríos et al. (2016) found that only 30.0% of medical students were willing to become live donors to an unrelated person, similar magnitude to our results (39.1%) [15]. A Croatian study by Topić et al. (2006) reported generally positive attitudes toward organ donation among sixth-year medical students, reflecting attitudes observed during the final stages of medical training [16].

This significant difference in willingness to donate while alive highlights an opportunity for targeted educational initiatives aimed at non-medical students. The main target of education should be the many students which remain undecided regarding living donation. Addressing misconceptions and highlighting the minimal impact of donation on the daily well-being of living donors could help increase willingness among students. Their hesitancy may stem from limited understanding of medical procedures, perceived health risks, or emotional and ethical concerns surrounding live donation.

Overall, our findings support a certain role of educational exposure in shaping positive attitudes toward organ donation. While medical education appears to increase willingness for living donation, the consistently high support for postmortem donation in both groups suggests that other influences—such as cultural values, religious beliefs, or altruistic tendencies—also contribute significantly to attitudes toward kidney donation.

### 4.2. Educational Background and Legal Awareness

Medical students demonstrated significantly greater awareness of Croatia’s organ donation laws compared to non-medical students (45.0% vs. 22.0%), likely due to exposure to health policies during their studies. Overall, more than half of medical students (55.0%) remained unaware of the legislation, which highlights a general deficiency in knowledge across both groups.

In addition to legal awareness, medical students also demonstrated slightly higher overall knowledge about kidney donation, including some basic facts, such as the ability to live with one functioning kidney, with 95.4% answering this correctly compared to 83.2% of non-medical students. These results emphasize the need to strengthen organ donation education within general curricula.

Notable gaps in knowledge were observed among medical students, a finding that warrants careful interpretation. Although medical students are generally exposed to specialized medical concepts during their training, participants in this study likely represented varying stages of education, including preclinical and early clinical years, during which formal instruction in transplantation medicine may be limited. For example, none of the 151 medical students selected the correct response range (91.0–100.0%) for the one-year kidney transplantation success rate. This result may reflect limited curricular exposure to transplantation outcomes at the undergraduate level or ambiguity in the wording and categorization of the questionnaire item rather than a true lack of knowledge. Taken together, these findings suggest that while medical education contributes to increased awareness of organ donation, it may not uniformly equip students with detailed understanding of long-term clinical outcomes of kidney transplantation. The reliance on social media as a primary source of information, as reported by many participants, further suggests that formal education on organ donation may not be reaching its full potential. These gaps highlight the need to amend curricular content by focusing even more on transplant education, as well as to launch broader public health initiatives to improve legal and clinical awareness among non-medical populations.

### 4.3. Religious and Cultural Influence

This study also aimed to explore whether religious beliefs influence either groups on their views on kidney transplantation. Most participants, regardless of their religious affiliation, stated that their beliefs did not influence their views on kidney donation. Religion can play a significant role in shaping attitudes toward organ donation, even when individuals do not explicitly recognize it as an influence. In countries such as Saudi Arabia and Pakistan, previous studies have shown that Islamic religious beliefs—particularly uncertainty about religious rules—can significantly influence willingness to donate [17,18]. Additionally, a study by Kobus et al. (2016) demonstrated that both age and religion have a considerable impact on attitudes toward organ transplantation, with younger participants and Catholics showing more favorable views compared to other religious groups and ages [19]. These findings suggest that Catholicism, which generally supports organ donation as an act of altruism and solidarity, may contribute to a cultural environment that is more accepting of transplantation.

In the Croatian context, where Catholicism is the predominant faith [20], such cultural norms may subtly reinforce positive attitudes toward donation, even among individuals who do not consciously attribute their beliefs to religion. Therefore, while most participants in this study stated that religion did not influence their opinions, it is likely that underlying cultural and religious values still play an indirect role in shaping their perspectives. Further research is needed to explore these findings in greater depth.

### 4.4. Source of Information

The survey also explored which source of information students use to gain knowledge about kidney donation. The results affirm social media as the most common and easily accessible source.

University students appear to have a concerning overreliance on a source that is well known to be full of misinformation, emotional narratives and conflicting messages, especially regarding topics like medicine. This is an important indicator that, in the future, low-quality information may contribute to increased doubt or fear of the transplantation process and a consequent decline in the rates of living organ donation.

Considering that social media is often associated with the spread of misinformation, its wide reach and accessibility make it a valuable tool for targeted educational campaigns. When used responsibly by governmental and health institutions, it can significantly improve university students’ understanding of transplantation and their willingness to support organ donation.

### 4.5. Concerns and Barriers to Organ Donation

Even though both groups of students showed willingness to donate their kidney, our study found that both groups expressed many concerns regarding donation. When asked to identify specific apprehensions, medical students most frequently cited “health risks”, “surgical complications” and concern about donation “interfering with their future plans”. Non-medical students expressed similar concerns but also emphasized their lack of knowledge about possible consequences (67.9%).

This suggests that even with proper, extensive medical understanding, the emotional and physical concerns remain. The persistence of these concerns indicates a need for a more meaningful approach when it comes to educating the public on transplantation. Campaigns should include testimonials from living donors, emotional reassurance, detailed explanations on the potential risks involved and how they can be prevented.

### 4.6. Recommendations and Implications

Our findings suggest that medical students demonstrated greater knowledge and willingness to donate while alive, likely due to their exposure to transplant-related education. Given this finding, it is evident that increasing both the quality and reach of educational initiatives is crucial to improve awareness and acceptance of kidney donation, particularly among non-medical students. We strongly believe that such educational programs should adopt an interactive and youth-centered approach, facilitated not only for students but also by students. Empowering young people to take the initiative in leading awareness campaigns may prove especially impactful, as peer-to-peer communication tends to be more genuine and engaging than formal messages delivered by institutions and authorities.

Social media is a major source of information regarding kidney transplant amongst our study’s participants. To address this, rather than dismissing social media, it should be utilized as a tool for spreading accurate, evidence-based content on transplantation. Students should take initiative in spreading accurate information, as they are more adept at using social media than older generations.

A model example of such student-led engagement can be found in an initiative called “Budi mRAK!”, which is part of the Croatian Medical Students’ International Committee (CroMSIC). This campaign has successfully promoted HPV vaccination and cervical cancer prevention through public outreach, education in schools, and interactive campaigns [21]. Inspired by their success, establishment of a similar student-driven organization or initiative focused specifically on organ donation awareness is recommended. For this group’s work to be effective and have an impact, it must be in constant communication with some form of mentor, collaborating with medical faculties, transplant centers, and public health authorities.

In addition, Croatia’s National Transplant Day, observed annually on the second Saturday in October, presents a valuable opportunity to increase public engagement. It is highly recommended that this day becomes more actively utilized for an interactive public outreach. These public health awareness days should be full of activities such as hosting informative booths, organizing creative workshops, distributing informative handouts, conducting street interviews, and initiating social media campaigns. These efforts would not only raise awareness but also potentially alleviate fears and misconceptions that often persist due to a lack of reliable information.

Medical students can play a key role in both the creation and delivery of educational content on organ donation. Their involvement would not only strengthen peer-to-peer learning but also enhance the credibility of public health campaigns. Additionally, by integrating awareness initiatives into nationally recognized events, Croatia can continue to build upon its strong foundation of organ transplantation. These efforts would help ensure that future generations remain informed, empathetic, and proactive in supporting this lifesaving practice.

To address apprehensions, it is recommended to incorporate real-life donor and recipient stories, hosting peer-led discussion circles, and organizing Q&A sessions with transplant professionals and individuals who have undergone the donation process.

Introducing structured education on transplantation within school curricula would contribute to early awareness. Incorporating a single, brief educational session into middle-school or high-school biology curricula, featuring living kidney donors or student volunteers, may empower young pupils and promote the development of positive attitudes toward organ donation from an early age.

Our study had certain limitations that should be acknowledged. This study relied on self-reported data, which may be influenced by social desirability bias. While the survey was broadly distributed, some degree of self-selection bias may still be present, as students with stronger opinions or prior interest in the topic may have been more likely to participate. A sample size calculation prior to the study was not performed and the findings should be interpreted within the exploratory and questionnaire-based design of the study. Analyses were primarily univariate and potential confounding demographic factors cannot be excluded. The unequal distribution of medical and non-medical students reflects enrollment patterns in Croatian university programs. The cross-sectional design precludes causal inference.

## 5. Conclusions

Both medical and non-medical students in this study demonstrated a high level of willingness to donate their kidneys posthumously, while medical students showed greater willingness to consider living donation. Non-medical students more frequently identified insufficient knowledge about the consequences of kidney donation as a major barrier against kidney donation. Social media remains one of the primary means through which students learn about kidney donation. Overall, these findings highlight differences in knowledge, risk perception and attitudes towards kidney donation between medical and non-medical students. Targeted educational interventions and public health campaigns may be beneficial in addressing misconceptions and could enhance awareness and foster more informed attitudes toward kidney donation and transplantation among future generations in Croatia.

## Figures and Tables

**Table 1:** Demographic data.

Participants Characteristics	Medical Students *N* (%)	Non-Medical Students *N* (%)	*p*-Value
Age			0.001
0–17	0 (0)	2 (0.4)	
18–21	73 (48.3)	353 (72.2)	
22–25	63 (41.7)	104 (21.3)	
25+	15 (10.0)	30 (6.1)	
Gender			0.280
Male	48 (31.8)	131 (26.8)	
Female	103 (68.2)	354 (72.4)	
Other	0 (0)	4 (0.8)	
Education program			0.001
Undergraduate (bachelor’s degree)	0 (0)	429 (87.7)	
Graduate medical degree—Preclinical years (1st–3rd year)	120 (79.5)	0 (0)	
Graduate medical degree—Preclinical years (4th–6th year)	31 (20.5)	0 (0)	
Postgraduate degree (master’s degree, PhD or equivalent)	0 (0)	60 (12.3)	
Religious affiliations			0.001
Christianity	81 (53.6)	334 (68.3)	
Islam	11 (7.3)	4 (0.8)	
Judaism	14 (9.3)	0 (0)	
Buddhism	1 (0.7)	0 (0)	
Agnostic	5 (3.3)	54 (11.0)	
None/Atheist	32 (21.2)	67 (13.7)	
Other	2 (1.3)	9 (1.8)	
Prefer not to say	5 (3.3)	21 (4.3)	

**Table 2:** Religious, medical and familial influences.

General Inquiry	Medical Students (%)	Non-Medical Students (%)	*p*-Value
Do your religious or cultural beliefs influence your views on kidney donation?			0.151
Yes	19 (12.6)	38 (7.8)	
No	117 (77.5)	389 (79.5)	
Not sure	15 (9.9)	62 (12.7)	
Do you have any known chronic health conditions?			0.300
Yes	21 (14.0)	50 (10.2)	
No	128 (84.7)	436 (89.2)	
Prefer not to say	2 (1.3)	3 (0.6)	
Do you have a family history of kidney disease?			0.001
Yes	13 (8.6)	67 (13.7)	
No	127 (84.1)	323 (66.0)	
Not sure	11 (7.3)	99 (20.3)	
Have you or someone you know donated or received a kidney organ?			0.837
Yes	19 (12.6)	58 (11.9)	
No	124 (82.1)	408 (83.4)	
Not sure	7 (4.6)	22 (4.5)	
Prefer not to say	1 (0.7)	1 (0.2)	

**Table 3:** Knowledge sources and awareness of kidney donation.

Knowledge About Kidney Donation	Medical Students *N* (%)	Non-Medical Students *N* (%)	*p*-Value
How did you learn about organ donation in Croatia?			
Social media (e.g., Facebook, Instagram, Twitter/X)	35 (23.2)	147 (30.1)	0.101
Internet Search (e.g., Google, Websites)	34 (22.5)	142 (29.0)	0.117
Printed Materials (e.g., Flyers, Posters, Brochures, Books, Textbooks)	25 (16.6)	75 (15.3)	0.718
Friends or Family	12 (7.9)	20 (4.1)	0.057
Healthcare Professionals (e.g., Doctors, Nurses)	71 (47.0)	99 (20.2)	0.001
Educational Campaigns or Events	28 (18.5)	76 (15.5)	0.382
News Articles (Online or Print)	18 (11.9)	87 (17.8)	0.089
Personal Experience (e.g., Knowing a Donor or Recipient)	18 (11.9)	49 (10.0)	0.505
Religious or Community Organizations	1 (0.7)	7 (1.4)	0.457
I have never gained adequate knowledge on the subject.	47 (31.1)	166 (33.9)	0.520
Tv Shows	0 (0)	5 (1.0)	0.597
School subjects and professors (Religion or Ethics course)	4 (2.6)	8 (1.6)	0.491

**Table 4:** Awareness of Croatia’s donation legislation.

Awareness	Medical Students *N* (%)	Non-Medical Students *N* (%)	*p*-Value
Did you know that in Croatia every citizen is by default an organ donor, unless they opt out?			0.001
Yes, I am familiar with this system	68 (45.0)	100 (20.4)	
No, I am not familiar with this system	83 (55.0)	389 (79.6)	

**Table 5:** Attitude regarding Croatia’s donation legislation.

Inquiry	Group	Strongly Disagree (%)	Disagree (%)	Neutral (%)	Agree (%)	Strongly Agree (%)	*p*-Value
What is your view on the Croatian default organ-donor system?	Medical students	5 (3.3)	9 (6.0)	16 (10.6)	30 (19.9)	91 (60.2)	0.001
	Non-medical students	35 (7.2)	44 (9.0)	109 (22.3)	105 (21.5)	196 (40.0)	

**Table 6:** Medical and non-medical students’ knowledge of kidney functions.

Inquiry	Medical Students *N* (%)	Non-Medical Students *N* (%)	*p*-Value
What do you think of the following statement: “A healthy person can survive with only one functioning kidney.”			0.001
True	144 (95.4)	402 (82.2)	
False	3 (2.0)	12 (2.5)	
Not sure	4 (2.6)	75 (15.3)	

**Table 7:** Stance of both groups on promoting kidney donation to others.

Encouraging Others	Medical Students (%)	Non-Medical Students (%)	*p*-Value
Would you ever encourage your family, friends, or peers to consider kidney donation?			0.001
Yes	33 (21.9)	80 (16.4)	
No	27 (17.9)	168 (34.4)	
Not sure	91 (60.3)	241 (49.3)	

**Table 8:** Opinion on whether medical professionals should be promoting kidney donation.

Inquiry	Group	Strongly Disagree (%)	Disagree (%)	Neutral (%)	Agree (%)	Strongly Agree (%)	*p*-Value
Kidney donations should be promoted by your physician.	Medical students	15 (9.9)	27 (17.9)	63 (41.7)	33 (21.9)	13 (8.6)	0.121
	Non-medical students	64 (13.1)	93 (19.0)	222 (45.4)	64 (13.1)	46 (9.4)	

**Table 9:** Medical and non-medical students’ view on ethically appropriate kidney donors.

Donor Type	Medical Students (%)	Non-Medical Students (%)	*p*-Value
Who do you ethically consider a suitable kidney organ donor?			
Deceased people (Over 65 years of age)	85 (56.3)	237 (48.5)	0.093
Deceased people (Under 65 years of age)	107 (70.9)	343 (70.1)	0.866
Healthy elderly (Over 65 years of age)	79 (52.3)	195 (39.9)	0.007
Healthy adults (Under 65 years of age)	121 (80.1)	343 (70.1)	0.016
Healthy young adults (Under 30 years of age)	107 (70.9)	270 (55.2)	0.001
Family and friends of the patient	129 (85.4)	389 (79.6)	0.108
People who are ‘brain-dead’ (with family consent)	107 (70.9)	268 (54.8)	0.001
Anyone who is willing	7 (4.6)	11 (2.2)	0.121

**Table 10:** Attitudes of medical and non-medical students toward different types of kidney donors.

Attitude Towards Different Types of Donations	Group	Strongly Negative (%)	Negative (%)	Neutral (%)	Positive (%)	Strongly Positive (%)	*p*-Value
My attitude towards living volunteer kidney donation.	Medical students	0 (0)	6 (4.0)	35 (23.2)	33 (21.9)	77 (51.0)	0.003
	Non-medical students	2 (0.4)	10 (2.0)	61 (12.5)	93 (19.0)	323 (66.1)	
My attitude towards volunteer deceased person kidney organ donation.	Medical students	2 (1.3)	3 (2.0)	23 (15.2)	23 (15.2)	100 (66.2)	0.671
	Non-medical students	9 (1.8)	10 (2.0)	58 (11.9)	95 (19.4)	317 (64.8)	
My attitude towards ‘brain-dead’ person kidney organ harvesting.	Medical students	2 (1.3)	10 (6.6)	28 (18.5)	32 (21.2)	79 (52.3)	0.521
	Non-medical students	13 (2.7)	23 (4.7)	113 (23.1)	105 (21.5)	235 (48.1)	

**Table 11:** Willingness to donate kidneys for various reasons and different circumstances.

Inquiry	Group	Strongly Disagree (%)	Disagree (%)	Neutral (%)	Agree (%)	Strongly Agree (%)	*p*-Value
I would be more willing to donate my kidney if I had more information about the kidney transplantation process and consequences.	Medical students	15 (9.9)	14 (9.3)	39 (25.8)	31 (20.5)	52 (34.4)	0.832
	Non-medical students	48 (9.8)	39 (25.8)	138 (28.2)	104 (21.3)	45 (29.7)	
I would be more willing to donate my kidney if a person I knew required a kidney transplant.	Medical students	7 (4.6)	10 (6.6)	15 (9.9)	39 (25.8)	80 (53.0)	0.689
	Non-medical students	14 (2.9)	24 (4.9)	54 (11.0)	141 (28.8)	256 (52.4)	
I would only potentially donate my kidney to a loved one or family member.	Medical students	16 (10.6)	16 (10.6)	39 (25.8)	38 (25.2)	42 (27.8)	0.875
	Non-medical students	44 (9.0)	56 (11.5)	112 (22.9)	125 (25.6)	152 (31.3)	
I would potentially donate my kidney to an unrelated person in need.	Medical students	21 (13.9)	39 (25.8)	44 (29.1)	31 (20.5)	16 (10.6)	0.472
	Non-medical students	86 (17.6)	115 (23.5)	165 (33.7)	79 (16.2)	44 (9.0)	
I would consider donating my kidney if I was in a coma/brain-dead.	Medical students	11 (7.3)	9 (6.0)	20 (13.2)	25 (16.6)	86 (57.0)	0.275
	Non-medical students	39 (8.0)	23 (4.7)	90 (18.4)	102 (20.9)	235 (48.1)	
I would consider donating my kidney for a sum of money/reward.	Medical students	58 (38.4)	36 (23.8)	29 (19.2)	16 (10.6)	12 (7.9)	0.011
	Non-medical students	142 (29.0)	93 (19.0)	138 (28.2)	42 (8.6)	74 (15.1)	

**Table 12:** Willingness to donate kidney(s) during lifetime and after death.

Donation During Lifetime and After Death	Medical Students (%)	Non-Medical Students (%)	*p*-Value
Would you consider donating a kidney while alive?			0.014
Yes	59 (39.1)	131 (26.8)	
No	16 (10.6)	69 (14.1)	
Not sure	76 (50.3)	289 (59.1)	
Are you willing to donate your kidneys after your death?			0.337
Yes	130 (86.1)	397 (81.2)	
No	3 (2.0)	18 (3.7)	
Not sure	18 (11.9)	74 (15.1)	

**Table 13:** Concerns and barriers expressed by medical and non-medical students.

Concerns/Barriers	Medical Students (%)	Non-Medical Students (%)	*p*-Value
My concerns (if any) around kidney donation:			
Health risks	116 (76.8)	385 (78.7)	0.619
Ethical/Religious concerns	8 (5.3)	20 (4.1)	0.526
Family objection	13 (8.6)	26 (5.3)	0.139
Lack of knowledge regarding the consequences	64 (42.4)	332 (67.9)	0.001
Surgery complications	110 (72.8)	370 (75.7)	0.485
Lack of compensation/Financial support	15 (9.9)	107 (21.9)	0.001
Affect it will have on my future plans.	82 (54.3)	233 (47.6)	0.153
Other	2 (1.3)	4 (0.8)	0.572

**Table 14:** Views on impact of kidney donation in personal lives.

Statements	Group	Strongly Disagree (%)	Disagree (%)	Neutral (%)	Agree (%)	Strongly Agree (%)	*p*-Value
Donating my kidney impacts my life in a positive way.	Medical students	14 (9.3)	32 (21.2)	71 (47.0)	26 (17.2)	8 (5.3)	0.354
	Non-medical students	37 (7.6)	79 (6.2)	276 (56.4)	74 (15.1)	23 (4.7)	
Donating my kidney impacts my life in a negative way.	Medical students	16 (10.6)	29 (19.2)	77 (51.0)	27 (17.9)	2 (1.3)	0.071
	Non-medical students	66 (13.5)	110 (22.5)	234 (47.9)	56 (11.5)	23 (4.7)	
I am worried about my family’s approval and objection regarding donating my kidney.	Medical students	57 (37.7)	27 (17.9)	39 (25.8)	18 (11.9)	10 (6.6)	0.044
	Non-medical students	227 (46.4)	107 (21.9)	103 (21.1)	33 (6.7)	19 (3.9)	
I am worried that kidney transplantation will leave me weak or disabled.	Medical students	9 (6.0)	31 (20.5)	44 (29.1)	46 (30.5)	2 (13.9)	0.643
	Non-medical students	30 (6.1)	75 (15.3)	150 (30.7)	153 (31.3)	81 (16.6)	

## Data Availability

The data presented in this study are available upon reasonable request from the corresponding author.

## Abbreviations

The following abbreviations are used in this manuscript:

CKD	Chronic kidney disease
ESRD	End-stage renal disease
HPV	Human papillomavirus
KRT	Kidney replacement therapy
Q&A	Question and answer
